# An Anomaly Node Detection Method for Wireless Sensor Networks Based on Deep Metric Learning with Fusion of Spatial–Temporal Features

**DOI:** 10.3390/s25103033

**Published:** 2025-05-12

**Authors:** Ziheng Wang, Miao Ye, Jin Cheng, Cheng Zhu, Yong Wang

**Affiliations:** 1 School of Information and Communication, Guilin University of Electronic Technology, Guilin 541000, China; 23022201005@mails.guet.edu.cn (Z.W.); yemiao@guet.edu.cn (M.Y.); acesohn@mails.guet.edu.cn (J.C.); 2 Information Center, Guilin Medical University, Guilin 541000, China; 3 School of Computer and Information Security, Guilin University of Electronic Technology, Guilin 541000, China; ywang@guet.edu.cn

**Keywords:** wireless sensor networks, anomaly detection, graph neural network, metric learning

## Abstract

Wireless sensor networks (WSNs) use distributed nodes for tasks such as environmental monitoring and surveillance. The existing anomaly detection methods fail to fully capture correlations in multi-node, multi-modal time series data, limiting their effectiveness. Additionally, they struggle with small sample scenarios because they do not effectively map features to classes. To address these challenges, this paper presents an anomaly detection approach that integrates deep learning with metric learning. A framework incorporating a graph attention network (GAT) and a Transformer is developed to capture spatial and temporal features. A novel distance measurement module improves similarity learning by considering both intra-class and inter-class relationships. Joint metric-classification training improves model accuracy and generalization. Experiments conducted on public datasets demonstrate that the proposed approach achieves an F1 score of 0.89, outperforming the existing approaches by 7%.

## 1. Introduction

Wireless sensor networks (WSNs) are self-organizing networks [1] consisting of wireless communication sensors that operate through multi-hop routing. They feature flexible network topology and the ability to form networks autonomously [2,3]. They can detect and perceive multiple modes of environmental information, such as temperature, humidity, and light intensity. They are extensively applied in various fields, including military, industrial inspection [4,5], intelligent agriculture [6], medical monitoring [7], and smart cities [8], among others.

Due to the use of wireless and multi-hop communication, WSN nodes are typically designed to be low-cost, compact, and low-power devices, which inherently limits their computing, memory, and communication capabilities. These constraints give rise to several disadvantages, such as narrow bandwidth, limited storage, short transmission range, and reduced sensing accuracy. Consequently, the reliability and energy efficiency of WSNs are often challenged [9,10]. During the process of data collection and transmission, such networks are also vulnerable to external intrusions and environmental disturbances, which may result in various anomalies in the data [11]. Abnormal or missing measurements [12] can also result from limited battery capacity, signal interference, software defects, and hardware-level node faults [13]. These issues may cause significant deviations from actual values. Therefore, the accurate detection and localization of abnormal data are critical for ensuring the stable and reliable operation of WSNs in practical applications.

The data collected by WSN nodes can be represented as time series data in mathematics, and multiple physical quantities collected by the same node correspond to multiple time series data, which is also called multi-modal data [14] in the literature. Not only will there be a correlation [15] between multi-modal time series data collected by the same node, but there will also be a correlation [16] between time series data collected by different sensor nodes. These correlations of sensor network data can be mathematically represented by attribute graphs, and the information of each attribute graph node corresponds to the multi-modal time series data collected by sensor nodes. The edge relationship between the nodes of the attributed graph is the connection relationship between the sensor nodes. Node anomalies in WSNs include point anomalies, context anomalies, and collective anomalies [17,18].

In the past, many researchers have attempted to address the problem of anomaly detection in WSNs using traditional algorithms, i.e., non-deep learning methods. When dealing with time series data in WSNs, traditional models, such as the Moving Average (MA) model, Autoregressive (AR) model, Autoregressive Moving Average (ARMA) model, and Autoregressive Integrated Moving Average (ARIMA) model, have been commonly employed. For example, in [19], the wavelet transform is used to decompose traffic data in the frequency domain, and a multi-level feature representation of the series is obtained through signal reconstruction. Additionally, sliding windows of different sizes are applied to observe characteristics of the sub-series at multiple scales. In [20], a spectral method is used for anomaly detection in time series data, where a high-pass graph filter extracts the high-frequency components of network signals, and anomalies are located by applying a threshold to specific frequency components.

However, traditional approaches struggle to comprehensively capture the spatio-temporal characteristics of both node attributes and network structure. The high diversity and complexity of spatio-temporal patterns in WSNs present significant challenges for accurate anomaly node detection.

In contrast, deep learning methods can effectively integrate network structure and node features, enabling the extraction of complex latent patterns [21,22]. For instance, ref. [23] employs a combination of Convolutional Neural Networks (CNNs) and Long Short-Term Memory (LSTM) networks to detect anomalies in WSNs. The model predicts future values in the time series and estimates the likelihood of anomalies at subsequent timestamps. Similarly, ref. [24] proposes an adversarial time series anomaly detection approach using a Variational Autoencoder (VAE) based on LSTM. In this method, the encoder transforms the input sequence into a latent representation, the generator reconstructs the original time series, and the discriminator identifies anomalies. Compared to traditional time series analysis methods, these deep learning approaches better capture temporal correlations but still struggle to effectively model spatial relationships between sensor nodes in WSN anomaly detection.

A favorable tool for extracting spatial correlation anomalies of WSN nodes is the anomaly detection method based on graph neural networks (GNNs). The authors of [25] propose a method that infers dynamic correlations between nodes using dynamic node-level input and fixed topology information. They further employ adaptive propagation to adjust the network structure and neighbor weights, enhancing the model’s ability to capture accurate feature information. The tGCN module designed by reference [26] combines GCNs and structural information, uses the hidden layer representation as the input of the decoder to obtain the reconstructed graph information, and calculates the reconstruction error to detect anomalies. The authors of [27] propose an anomaly detection method combining a GCN and non-negative matrix factorization. This method uses the information on neighbor nodes to extract the feature expression of each node and complete matrix factorization, and then performs similarity grouping by multi-layer Perceptron (MLP). The authors of [28] propose an end-to-end anomaly edge detection framework based on an extended temporal GCN and GRU with an attention mechanism. Some researchers use anomaly detection methods based on distance measurement. The authors of [29] use reconstruction to obtain data information similar to the original data after decoding; then, they preset a feature center for clustering, map the normal samples in the data to the location closer to the feature center, and map the abnormal samples to the location far from the feature center so as to train the network. However, this scheme only considers the degree of similarity between positive and negative samples. In [30], a fully convolutional network is used to extract the feature information of data; then, the distance between a target sample and a normal sample is calculated as the standard to evaluate the abnormal degree of the sample. Metric learning is used for modeling and classification. However, these methods have limitations. Traditional metric learning mostly uses linear mapping for similarity modeling, which has difficulties in capturing the complex feature relationships of high-dimensional nonlinear data; especially in small sample scenarios, the correspondence between feature representation and classification target is prone to deviation. Secondly, current similarity learning methods based on the comparison of positive and negative examples mainly focus on the binary discrimination of sample pairs. However, they lack a multi-level comparative analysis of intra-class similarity and inter-class difference, which leads to insufficient sensitivity to subtle feature changes. These problems restrict the improvement in anomaly detection accuracy and model generalization ability.

Based on the existing research on WSN anomaly detection, this paper aims to address the problems and limitations of the existing literature and carries out the following work:

(1) This paper proposes an anomaly detection framework that integrates metric learning with deep learning. Unlike traditional metric learning methods, which typically rely on linear mapping to assess sample similarity, deep learning leverages nonlinear feature extraction to achieve more precise similarity measurements for high-dimensional data. Compared with traditional deep learning methods, this method can autonomously learn the similarity information for anomaly detection tasks and solve the problem that it is difficult to obtain accurate correspondence between feature information and information classification in small sample data. The accuracy and generalization ability of the model for anomaly detection tasks are improved.

(2) Compared with the existing methods that use positive and negative examples to obtain similarity, which lack the comparison learning of intra-class and inter-class similarity relationships, this paper uses triplet loss for metric learning. By comparing the similarity of paired samples, the model can learn subtle features better during training and improve the ability of the model to identify abnormal node information.

## 2. Related Definitions and Techniques

### 2.1. Definition of Anomaly in WSN Anomaly Detection Problem

When the WSN is affected by the external environment, such as fire, earthquake, air pollution, or hardware or software failures, including insufficient battery, signal interference, software defects, and so on, the measured data of the sensor node will deviate from the real data, that is, the data are abnormal [31]. The data collected by the WSN nodes and the relationship between the nodes can be represented by an attributed graph. The node information of each node is the multi-modal time series data collected by the sensor nodes, and the connection relationship between the sensor nodes corresponds to the side information in the graph data. Generally, for the multi-modal time series data collected by sensor nodes, the anomaly detection of node information includes the point anomaly, context anomaly, collective anomaly, and correlation anomaly [32,33] of single time series data. Point anomaly refers to the normal data in single time series data that significantly deviate from the original dataset. The large difference between the expression value in a certain scenario or time period and the previous time period is referred to as a context anomaly. This local anomaly can reflect the ability of an anomaly detection model to learn context information, which is more challenging. The exception set of a single data point is called a collective or population exception, where a single data point does not necessarily have an exception, but when a collection of multiple similar data occurs, it is regarded as a collective exception.

The data collected by the nodes of the sensor network can be mathematically represented as time series data over time, and the multiple physical quantity data collected by each sensor node corresponds to multiple time series data, which is also called multi-modal data [14,15]. It is known that not only the multi-modal time series data collected by the same node may be correlated with each other (which is a kind [16] of temporal correlation), but also the time series data collected by different sensor nodes may be correlated with each other (which is represented as the spatial location correlation [16] of nodes).

**Figure 1:**
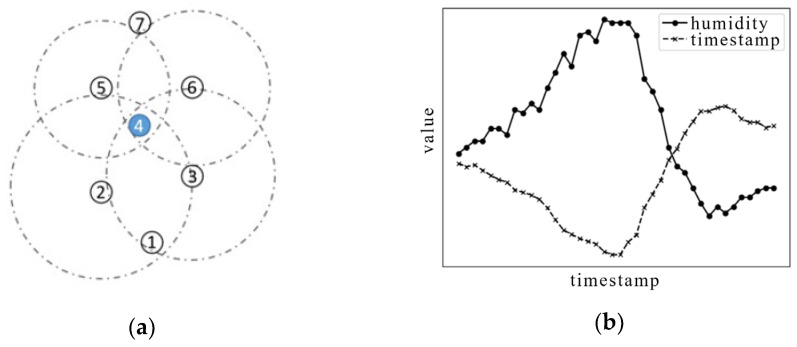
(**a**) Spatial location correlation example of nodes. (**b**) Multi-modal data synchronization change in WSNs.

Figure 1a shows the spatial position of some nodes. It is easy to find that the distance between node 4 and nodes 2, 3, 5, and 6 is relatively close. When the humidity and other data of the four surrounding nodes increase, the relevant value of node 4 will be affected to a certain extent. Temporal correlation means that the data of different modes will affect each other and be correlated in the process of changing with time. That is, the change in data information in a certain mode will affect the change trend in the data information in other modes. Figure 1b shows the synchronous change in humidity and temperature in the time series data of a WSN. Under normal circumstances, as the temperature rises, the water vapor pressure in the air will increase when the water vapor reaches saturation, and the relative humidity will decrease; that is, the temperature and humidity are inversely proportional. When there is a violation of these normal correlations, it means that there is an abnormal correlation in the sensor network.

### 2.2. Anomaly Detection Problem Description

In WSNs, each sensor node can be represented as a node in a graph, and the network topology corresponds to the edges between these nodes. Therefore, a WSN can be modeled as an attributed graph G=(A, X), where A is the adjacency matrix obtained by the topology structure of the attribute graph G. When the edge connecting the *i*-th node and the *j*-th node exists, $A_{ij}=1$; on the contrary, when the edge connecting the *i*-th node and the *j*-th node does not exist, $A_{ij}=0$. The attribute matrix of attributed graph G contains multi-modal and multi-node temporal information in sensor networks. $X\in R^{N\times D}$, where N is the number of nodes included in the network and D is the dimension of each node’s attribute feature vector.

The output label of the WSN anomaly detection model at time t is as follows.

(1) $$y_{t}=F(X_{t},\varpi )$$

where $y_{t}$ is the output label at time t, F is the mapping function of the anomaly detection model, and ϖ is the parameter of the anomaly detection model. When the output label is 1, there is an anomaly at time t; when the output label is 0, it is normal at time t. Sensor nodes collect data at the same time point t and transmit them to the sink node, which is responsible for storing data from all nodes at the same time. This synchronization mechanism ensures that the data at the same time point can be processed together, enabling the detection of anomalies in the corresponding attribute graph of the WSN. The method then determines whether there is a spatio-temporal correlation anomaly in the WSN and the correlation anomaly between multi-modal time series data. The text detects the anomaly of the corresponding attribute graph of the WSN to determine whether there is a spatio-temporal correlation anomaly in the WSN and the correlation anomaly between multi-modal time series data.

### 2.3. Artificial Neural Network

As a complex and powerful network structure, artificial neural networks (ANNs) are widely used to solve various practical problems, which often involve the processing of multiple nodes and multiple output points. Compared with the advantages of the human brain in parallel information processing, ANNs are more inclined to adopt a linear thinking mode for modeling. This way of thinking enables ANNs to perform better than humans in serial arithmetic tasks by performing fast and accurate sequential numerical calculations.

The nonlinear characteristics of ANNs give them the ability to perform complex logic operations and realize nonlinear relationships, which makes them a powerful tool for dealing with various complex problems. The whole network is composed of a large number of nodes (or neurons) connected to each other, where each node represents a specific output function, that is, an activation function. The connection weights between nodes reflect the degree of association between them, and these weights are crucial for an ANN to simulate human memory. The final output of the network is not only affected by the network structure and connection mode but also by the comprehensive adjustment of weights and activation functions, which makes an ANN adapt better to the needs of different problems and provide more accurate output results.

**Figure 2:**
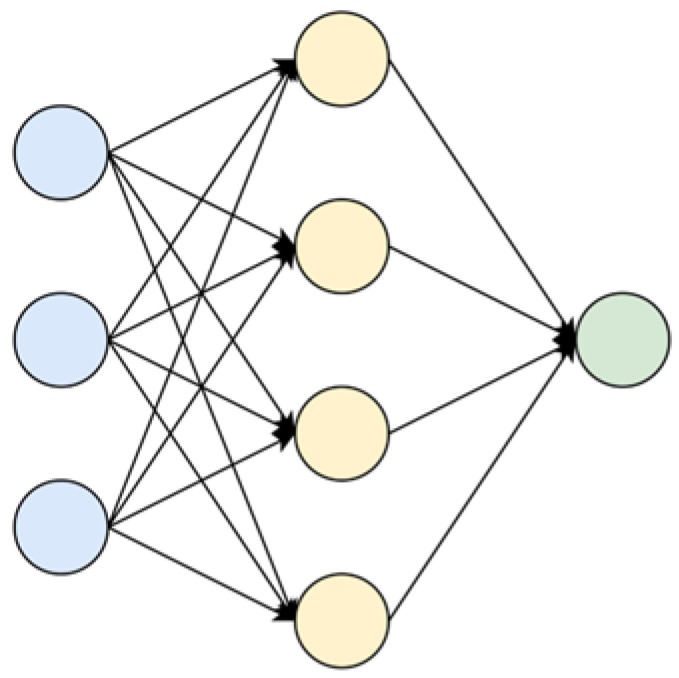
Artificial neural network structure diagram.

As shown in Figure 2, an artificial neural network usually consists of an input layer, a hidden layer, and an output layer; different from the input layer and the output layer, the number of hidden layers is not limited to one. The input layer is used to obtain data information from outside the network, such as graph data information, timing information, user information, etc. The hidden layer is used to analyze and learn the data information, and the output layer is used to generate corresponding single or multiple output results. According to the complexity of the problem, we can decide whether we need to add more than one hidden layer.

With the development of ANNs, neural networks have been widely used for time series data and graph data. The following is an introduction to some related artificial neural networks.

#### 2.3.1. Graph Convolutional Neural Networks

Traditional models, such as CNNs and RNNs, are usually used to extract the image feature information in image recognition or the information in natural language sequences. However, traditional methods do not make full use of the adjacency matrix and cannot combine the structural information in graph data well. A graph convolutional network (GCN) can associate the attribute information and topological information in graph data and realize the end-to-end learning of the inherent attributes of the objects in the graph and the topological information between the objects, which can affect the extraction of graph feature information and the learning of graph information at the same time. GCNs have better adaptability for graph learning tasks. For a GCN with L layers, the feature of the *i*-th node $v_{i}$ in the *l*-th layer is denoted by ${h_{i}}^{\left(l\right)}$, the feature of the l layer is denoted by $H^{\left(l\right)}=\{{h_{1}}^{\left(l\right)},\;{h_{2}}^{\left(l\right)},\;\ldots ,{h_{N}}^{\left(l\right)}\}$, and N is the number of nodes. The input of each layer is the adjacency matrix and the feature representation [34,35] of the previous layer. Then. the inter-layer propagation mode is as follows.

(2) $$H^{\left(l\right)}=\Phi (AH^{\left(l-1\right)}W^{\left(l-1\right)})$$

where $H^{\left(l\right)}$ and $H^{\left(l-1\right)}$ are the feature representations of layer l and layer l−1, respectively, A represents the adjacency matrix of the graph, W represents the weight matrix, Φ( ) is the activation function, $H^{\left(0\right)}=X$, and X is the attribute matrix.

In order to avoid the problem of changing the distribution of feature information and strengthen the data stability during network learning, the adjacency matrix can be normalized. At the same time, in order to retain the feature information of the node itself, the adjacency matrix can be normalized, and the self-connection of the node can be added to the original adjacency matrix. Then, the graph convolutional layer can be expressed as follows.

(3) $$H^{\left(l\right)}=\emptyset \left({\tilde{D}}^{-\frac{1}{2}}\tilde{A}{\tilde{D}}^{-\frac{1}{2}}H^{\left(l-1\right)}W^{\left(l-1\right)}\right)$$

where $\tilde{A}=A+I$, $\tilde{D}$ is the corresponding degree matrix of $\tilde{A,}$ and:

(4) $${\tilde{D}}_{ik}=\left\{\begin{array}{cc} \sum_{j}{\tilde{A}}_{ij}, & i=k\\ 0, & i\neq k \end{array}\right.$$

#### 2.3.2. Temporal Convolutional Networks

A temporal convolutional network (TCN) is a convolutional network algorithm for time series processing tasks. Based on the traditional algorithm, TCNs enlarge the receptive field, support parallel computing ability, and effectively solve the lack of memory retention and gradient explosion or disappearance. A time series of time length T is set. In traditional convolution methods, the length of the input sequence is limited by the size of the convolution kernel. As a convolutional network algorithm for time series processing tasks, temporal convolutional networks use dilated convolution to solve the problem that the length of the input sequence is limited by the size of the convolution kernel [36,37]. The expression formula is:

(5) $$F(t)=\sum_{i=0}^{k-1}f(i)\cdot x_{t-d\cdot i}$$

where d is the expansion coefficient, k is the size of the convolution kernel, and filter $F\left(t\right)=(f_{1},f_{2},\ldots ,f_{i})$. Then, the size of the expansion between adjacent sampling points is d−1. The receptive field size S after expansion is:

(6) S=K+(d−1)×(k−1)=d×(k−1)+1

According to the formula, to obtain a larger receptive field, the size of the convolution kernel can be increased, or the expansion coefficient can be increased to increase the distance between the sampling points.

In TCNs, sequence information can be transmitted through cross-layer, where the transmission mode can be expressed as follows.

(7) Z(x)=Relu(F(x)+x)

where Z is the output of the entire convolutional network. In order to ensure that the size of the input and output in the network module matches, an additional convolutional layer can be used to process the sequence x to complete the cross-layer transmission.

#### 2.3.3. Graph Attention Network

A graph attention network (GAT) is a graph neural network based on an attention mechanism. Different from GCNs and the other graph neural networks mentioned above, a GAT uses node features for similarity calculation and fully considers the correlation information between the target node and its neighbor nodes. At the same time, when using a GAT, node-level tasks do not need to provide complete graph structures in advance. When dealing with different sizes of the neighborhood domain and different node distributions, neighbor weights are of great importance [38].

For a GAT with L layers, the feature of the *i*-th node $v_{i}$ at the l layer is denoted by $h_{i}^{\left(l\right)}$, the feature of the l layer is denoted by $H^{\left(l\right)}=\left\{{\vec{h}}_{1}^{\left(l\right)},{\vec{h}}_{2}^{\left(l\right)},\ldots ,{\vec{h}}_{N}^{\left(l\right)}\right\}$, ${\vec{h}}_{i}^{\left(l\right)}\in R^{F^{\left(l\right)}}$, and N is the number of nodes. The output after the attention layer of the graph is $H\prime^{\left(l\right)}=\left\{\vec{h}\prime_{1}^{\left(l\right)},\vec{h}\prime_{2}^{\left(l\right)},\ldots ,\vec{h}\prime_{N}^{\left(l\right)}\right\}$, $\vec{h}\prime_{i}^{\left(l\right)}\in R^{F\prime^{\left(l\right)}}$.

Firstly, the input graph information is transformed into higher dimensional feature information, that is, the information $h_{i}$ of node i is transformed. To initialize a weighting matrix and map the nodes characteristic dimension F to dimension F′, the self-attention on each node in the graph is used to calculate weight concentration between any two nodes. The importance of node j to node i is computed as follows:

(8) $$e_{ij}=a\left(Wh_{i},Wh_{j}\right)$$

where mapping $a:R^{F}\times R^{F^{\prime }}\to R$ is used to combine the extracted high-dimensional feature information through a concatenation operation and then correspond it to the low-dimensional information. $e_{ij}$ represents the correlation coefficient between node i and node j.

Secondly, for the sake of the correlation coefficient of different nodes in the same order of magnitude range and to improve the comparability between data, regularization processing and the Softmax coefficient are used to obtain attention.

(9) $$\alpha_{ij}=\frac{exp\left(e_{ij}\right)}{\sum_{k\in N(i)}exp\left(e_{ik}\right)}$$

where N(i) indicates the neighbor node of node i. The attention mechanism is a single-layer feed-forward neural network using the LeakyReLU activation function. Then, the attention coefficient can be expressed as follows.

(10) $$\alpha_{ij}=\frac{exp\left(LeakyReLU\left({\vec{a}}^{T}\left[W{\vec{h}}_{i}\|W{\vec{h}}_{j}\right]\right)\right)}{\sum_{k\in N(i)}exp\left(LeakyReLU\left({\vec{a}}^{T}\left[W{\vec{h}}_{i}\|W{\vec{h}}_{k}\right]\right)\right)}$$

where ‖ indicates the concatenation operation. Finally, based on the attention given to the normalized coefficient and the characteristics of the linear combination of the corresponding processing, the target node’s corresponding output characteristic information is determined. The formula is as follows:

(11) $${\vec{h}}_{i}^{\prime }=\sigma \left(\sum_{j\in N(i)}\alpha_{ij}W{\vec{h}}_{j}\right)$$

In order to make the GAT more stable, based on the original network, an increased attention mechanism is used to obtain the output characteristic information.

**Figure 3:**
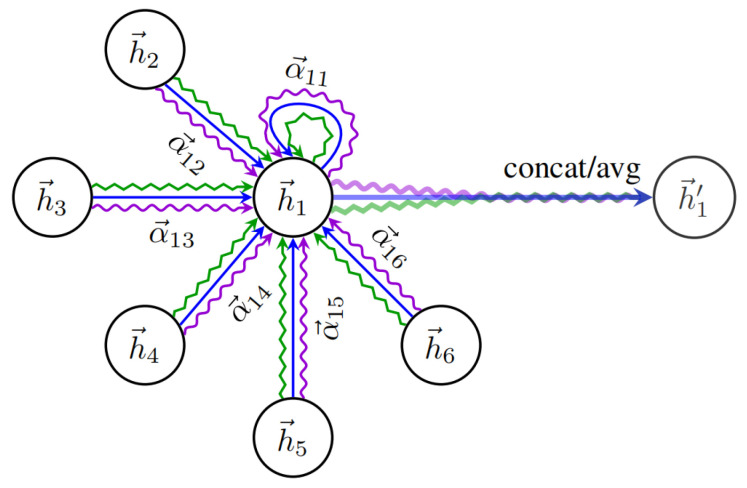
Nodes of the bull attention mechanism.

**Figure 4:**
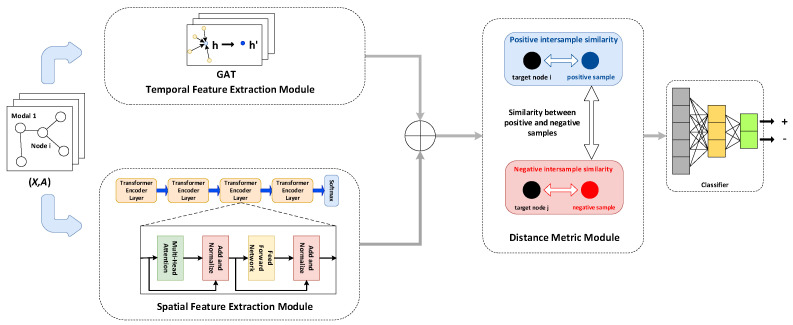
ST—DMLAD model framework.

In Figure 2, Figure 3 and Figure 4, the three colored curves represent three different heads. Under the different heads, nodes can learn different characteristics; these characteristics are used to join the nodes together to obtain the output information. The computation formula using the K head attention mechanism is as follows:

(12) $${\vec{h}}_{i}^{\prime }={\|}_{k=1}^{K}\sigma \left(\sum_{j\in N(i)}\alpha_{ij}^{k}W^{k}{\vec{h}}_{j}\right)$$

### 2.4. Metric Learning

Many algorithms in machine learning need to use distance as a metric to measure the similarity degree of the feature vector. Based on the complete data classification, these methods include clustering, dimension reduction, and anomaly detection, such as similarity search tasks and k-means (K-means algorithm, K nearest neighbor algorithm, density clustering algorithm, etc.).

The purpose of metric learning is to obtain a metric matrix that can effectively represent the similarity between data samples through training and learning. During training, the distance between samples of the same class is reduced or limited, and the distance between samples of different classes is increased, so that the same class samples in the new feature space are more compact and the different class samples are more distant. The parametric mapping function from features to classes is learned autonomously from limited samples, which improves the ability of the model to distinguish samples from different classes.

Traditional deep learning methods do not work well when the number of samples in a class is small. A measurement study can solve this problem. The common method is to use deep learning to extract the feature information of the original data, map it to the Euclidean space, and then train the model to make the distance between samples of the same class small and the distance between samples of different classes large [39].

For two input samples, the training process is as follows:

(13) $$L={\mathrm{yd}}_{a,b}^{2}+(1-y)max{\left(margin-d_{a,b},0\right)}^{2}$$

where y is the label of the pair of samples. When y=1, the two samples belong to the same class; conversely, when y=0, the two samples belong to different classes. $d_{a,b}$ refers to the Euclidean distance between the two samples. margin is the threshold parameter.

On this basis, the similarity relationship of sample pairs in the same class can be further considered. Three samples are needed to calculate the loss, which are called anchor samples, positive samples, and negative samples. Among them, the anchor sample is the sample we focus on, the positive sample and the anchor sample have the same class label, and the negative sample and the anchor sample have different class labels.

Suppose that the feature information expressions of the three samples are A (anchor sample), P (positive sample), and N (negative sample). Then, the form of the loss function is:

(14) $$L=max(d_{A,P}-d_{A,N}+margin,0)$$

## 3. WSN Anomaly Node Detection Method Based on Deep Metric Learning and Spatio-Temporal Feature Fusion

### 3.1. Deep-Metric-Learning-Based Anomaly Node Detection Model in WSNs

The framework of the designed anomaly detection model (ST-DMLAD) is shown in Figure 4. The model primarily consists of three modules: a feature extraction module, a distance measurement module, and a classification module.

The model takes both the attribute information and structural information of the wireless sensor network as input, specifically the attribute matrix and adjacency matrix. These matrices are fed into a feature extraction module composed of a spatial feature extractor and a temporal feature extractor. After extracting spatial and temporal features, the model performs feature fusion and dimensionality reduction to obtain spatio-temporal feature representations of the wireless sensor network. These fused features are then passed to the distance metric module, which computes sample similarity. Based on the similarity information, metric learning is performed to learn discriminative similarity features. Finally, the classification module identifies anomalous node information.

The detailed description of each module in the proposed anomaly detection model framework is outlined below.

### 3.2. Feature Extraction Module

The feature extraction module performs feature extraction by space and time by joining together the characteristics of the two modules’ output information to obtain the characteristics of space and time information for the matrix shape [N, M, 2 W], where N represents the number of nodes in the WSN, M denotes the number of modalities in the WSN, and W is used for processing the temporal data length of the WSN. Then, the space–time characteristic information matrix undergoes a dimension reduction operation, where the shape of the matrix after dimension reduction is [N, M, W], which is the output of the feature extraction module.

The specific content and structure of the spatial feature extraction module and the temporal feature extraction module are described below.

#### 3.2.1. Spatial Feature Extraction Module

The spatial feature extraction module uses the GAT to extract the spatial feature information of the WSN time series data. The spatial feature information captures the spatial relationship between the target node and its neighboring nodes. The spatial feature extraction module receives as input both the attribute matrix and the adjacency matrix of the WSN. The shape of the attribute matrix X is [N, M, W], and the shape of the adjacency matrix A is [N, N]. In this paper, the Top-k nearest neighbor method is used to obtain the adjacency matrix of the WSN. That is, for the target node $v_{i}$, the set of the Top-k nearest nodes is $N(v_{i})$, where $v_{j}\in N(v_{i})$. Then, node $v_{i}$ and $v_{j}$ are regarded as connected, i.e., $A_{ij}=1$. On the contrary, if node $v_{i}$ and $v_{j}$ are not connected, i.e., $A_{ij}=0$.

As can be seen from the previous content, for the target node $v_{i}$, its feature representation can be obtained as:

(15) $$X_{i}=\sigma \left(\sum_{j\in N(v_{i})}\alpha_{ij}WX_{j}\right)$$

where $\alpha_{ij}$ is the attention coefficient between nodes $v_{i}$ and $v_{j}$ and W is the weight matrix.

After adding the multi-head attention mechanism on this basis, the obtained features are expressed as follows.

(16) $$X_{i}={\|}_{k=1}^{K}\sigma \left(\sum_{j\in N(v_{i})}\alpha_{ij}^{k}W^{k}X_{j}\right)$$

The output is the spatial feature information matrix, whose shape is [N, M, W].

#### 3.2.2. Temporal Feature Extraction Module

This article designs a temporal feature extraction module based on the Transformer encoder layer to extract time-based characteristics from WSN time series data. The input of the temporal feature extraction module is the attribute matrix X of the WSN. As illustrated in Figure 4, the attribute matrix passes through four Transformer encoder layers, where each layer is encoded by a long attention mechanism, the feedforward neural network, and the residual connection. The output of multi-head attention is:

(17) $$X_{MHA}=MultiHead(Q,K,V)=\;\mathrm{Concat}\;\left({head}_{1},\ldots ,{\;\mathrm{head}\;}_{h}\right)W^{O}$$

The output of the Transformer encoder layer after layer l is:

(18) $$X_{trans}^{\left(l\right)}=\;\mathrm{TransfomerEncoderLayer}\;(X^{\left(l-1\right)})$$

$X^{\left(0\right)}$ is the time feature extraction module matrix X input attribute.

After the output of the feature extraction module passes through the Softmax function, the resulting time feature information matrix has the shape [N, M, W]. It is then combined with the spatial feature information matrix, which undergoes dimensionality reduction. The shape of the resulting dimensionality-reduced feature information matrix is also [N, M, W].

### 3.3. Distance Metric Module

The distance metric module aims to learn the similarity between samples and differentiate positive sample pairs from negative ones by forming sample pairs. The distance measurement module uses the feature extraction module matrix as the input output characteristics of space and time information. For the target sample $v_{i}$, the trained model puts the target sample closer to the positive samples of the same category and farther away from the negative samples of different categories. In this process, the target sample, positive sample, and negative sample are regarded as a triple $\left(x^{a},x^{p},x^{n}\right)$, and the positive sample pair $\left(x^{a},x^{p}\right)$ and negative sample pair $\left(x^{a},x^{n}\right)$ are constructed, where $x^{a}$ denotes the target sample, $x^{p}$ denotes the positive sample, and $x^{n}$ denotes the negative sample. Then, the model is trained so that the distance between the sample points with the same class is close enough and the distance between the sample points with different classes is far enough; that is, the distance between the target sample $x^{a}$ and the positive sample $x^{n}$ is much smaller than the distance between the target sample $x^{a}$ and the negative sample $x^{n}$. This process can be expressed as follows.

(19) $$\begin{array}{c} {\left\|f\left(x^{a}\right)-f\left(x^{p}\right)\right\|}_{2}^{2}+\theta <{\left\|f\left(x^{a}\right)-f\left(x^{n}\right)\right\|}_{2}^{2}\\ \forall \left(f\left(x^{a}\right),f\left(x^{p}\right),f\left(x^{n}\right)\right)\in T \end{array}$$

where θ is the distance between positive and negative sample pairs and T is the selected triple in the dataset.

To learn more about the model anomaly information of different situations, this article designs a variety of abnormal information used in the injection way to enrich a triple loss to the negative samples. This part of the specific operation can be artificial injection in the experimental part of the exception-information-related introduction.

### 3.4. Classification Module

Following metric learning in the distance measurement module, the information matrix is categorized, with the target node being classified as either normal or abnormal based on similarity. Specifically, the multi-layer fully connected layer is used to map the feature information into high-dimensional and low-dimensional spaces for classification.

The process can be represented as:

(20) p=Softmax(f(f…f(X)))

where p is the classification probability that the target node is judged as normal or abnormal.

### 3.5. Loss Function

This section presents the model design, which utilizes two types of loss functions: triple damage and loss. After combining these two loss functions, they are used together as the loss function to train the model. The designed loss function is as follows:

(21) $$L_{triplet}=max\{d(a,p)-d(a,n)+margin,0\}$$

(22) $$L_{discrim}=-[plogp+(1-p)log(1-p)]$$

(23) $$Loss=L_{triplet}+L_{discrim}$$

where Loss is the loss function used in the model designed in this paper, $L_{triplet}$ is the triplet loss function, and $L_{discrim}$ is the classification loss function.

### 3.6. Experimental Results and Analysis

This section mainly describes the relevant experiments performed to verify the performance of the model designed in this section. The content includes the real dataset used in the experiment and the processing of the dataset, the method of manual exception injection, the indicators used to evaluate the performance of the model, and the relevant comparison experiments. Linux system version 5.4.0-148-generic was used to run the relevant code. Two CPUs were used in the server model: Intel^®^ Xeon^®^ Gold 5218 @ 2.30 GHz (Intel Corporation, Santa Clara, CA, USA), with a memory size of 125 GB; and the GPU was an NVIDIA GeForce RTX 3080 (NVIDIA Corporation, Santa Clara, CA, USA), with CUDA version 11.7. The code is in the python language, and the versions of the related module packages used are shown in Table 1.

#### 3.6.1. Experimental Datasets

The real dataset used in this section is the WSN dataset collected by Intel Berkeley Research Lab field deployment. These data include the 28 February 2004 solstice on 5 April, which were obtained from the deployment-related sensor locations of the Intel Berkeley Research Lab.

**Figure 5:**
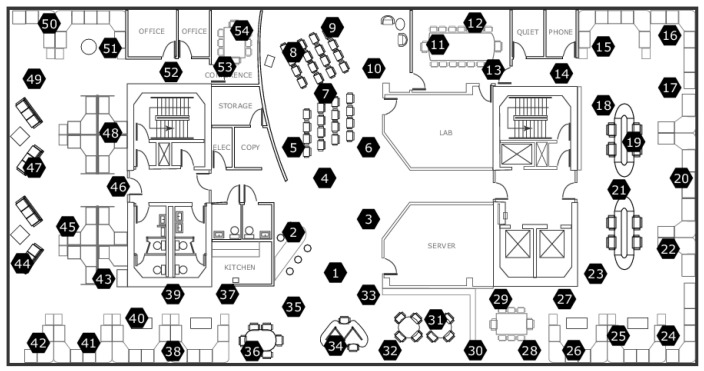
Map of sensor spatial location distribution for the WSN dataset collected by Intel Berkeley Lab field deployment.

The dataset includes humidity, temperature, light, and voltage values collected by 54 sensors during that time period. Each sensor collects relevant environmental information every 31 s, and a TinyDB network query processing system based on the TinyOS platform is used to collect data. The spatial location distribution map of the 54 sensors is shown in Figure 5.

After processing the original dataset, the relevant node information with missing information or an obvious abnormal information record was analyzed. Therefore, the dataset used in this section is composed of partial data selected from the original dataset. The experimental dataset includes 51 sensor nodes, 12,900 moments, and the sensor data of three modes: humidity, temperature and voltage value. The Top-k nearest neighbor method was used to process the position coordinates of the sensors recorded in the dataset to generate its topology.

#### 3.6.2. Manual Injection of the Abnormal Mode 

This section introduces the method of injecting anomaly information into the experimental dataset. The injected anomaly information specifically includes five different kinds of anomaly information: point anomaly, context anomaly, collective anomaly, spatial correlation anomaly, and temporal correlation anomaly. In the experiment, different types of anomalies were randomly selected to inject abnormal information, and the injected nodes, modes, and moments were randomly generated within a certain range.

- Point anomalies.

A point anomaly refers to the data information of a single data point that is significantly different from other data information. In this section, a number of time points are randomly selected in the specified time window of the injection anomaly, and the point anomaly is injected by scale transformation. The specific transformation method of the data at time t is expressed as follows.

(24) $$X_{t}^{point}=(1+a)*X_{t}$$

where α is the multiple of the scale transformation; in this experiment, α∈{0.3,0.5,0.8}.

**Figure 6:**
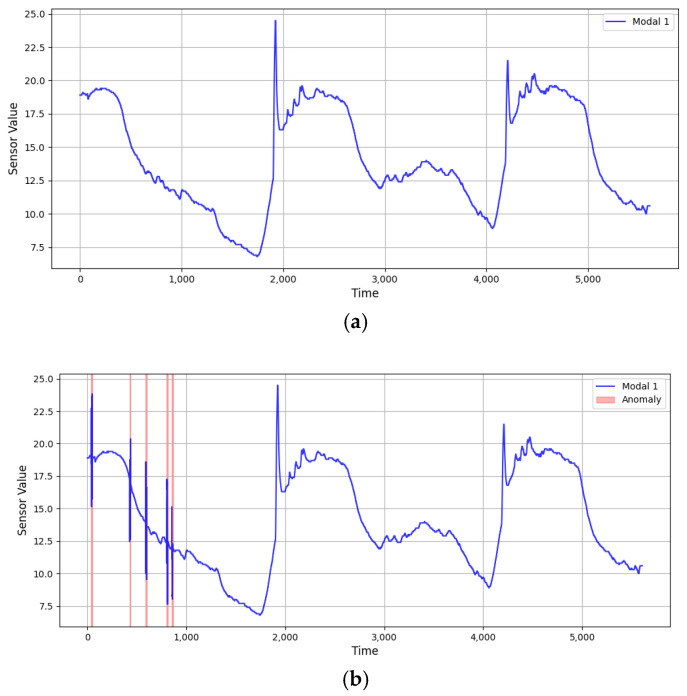
(**a**) Original data before point anomaly injection. (**b**) Data after point anomaly injection (red shaded area indicates the injected anomaly).

In Figure 6, the graph on the left does not include injection point abnormal data. The graph on the right includes a little bit of abnormal data, where we can obviously see an abnormal moment within the other data.

- Context anomalies.

A context anomaly mainly refers to an abnormal sample whose value is normal, but it exists in the context environment. The injection of context anomaly information is divided into two types: upward trend and downward trend. That is, there is time t in the time window, including:

(25) $$X_{t}^{Cont}=X_{t-1}\pm \beta (X^{max}-X^{min})$$

where $X^{max}\;and\;\;\;X^{min}$ refer to the maximum and minimum values of the data in the time window, respectively. t∈[t,t+τ], where τ is the window length of the injected abnormal information with an upward or downward trend, and β is the proportion coefficient of the context anomaly offset.

**Figure 7:**
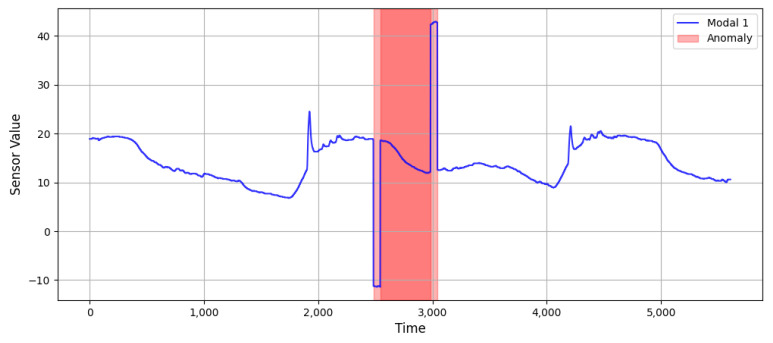
Data after contextual anomaly injection (red shaded area indicates the injected anomaly).

Figure 7 illustrates the context of rising trends in anomaly-information-injected data in comparison with the original data. The original data are shown on the left, and the data with anomalies injected are shown on the right.

- Collective anomalies.

**Figure 8:**
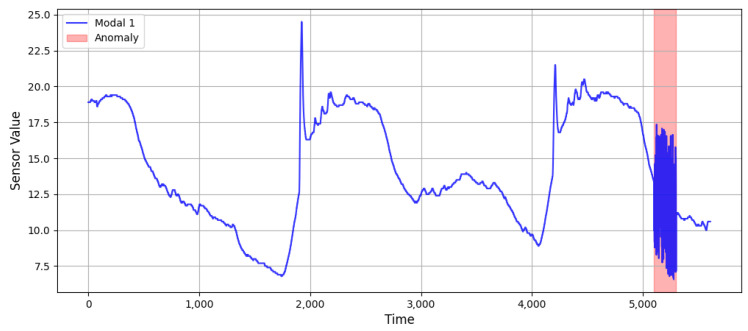
Data after collective anomaly injection (red shaded area indicates the injected anomaly).

In Figure 8, the red part shows the collective abnormal information injected manually. A collective anomaly refers to multiple data that collectively cause an anomaly. In collective anomalies, single data information cannot be regarded as abnormal information, but a set of multiple data information can produce an anomaly. Collective exceptions are injected as shown in the following formula:

(26) $$X_{t,t+\tau }^{Coll}=a\times sin\theta +b$$

where t∈[t,t+τ] and τ are the length of the window in which the collective exception information is injected. Both a and b are constants.

- Spatial correlation anomalies.

There is correlation information between sensor nodes and their neighbor nodes. First, the neighbor node j of target node i is obtained through the topology structure, and j∈N(i), where N(i) is the neighbor node set of target node i. The spatial correlation between target node i and neighbor node j is calculated. In this section, we use the pearson correlation coefficient to calculate the correlation coefficient, which is calculated as follows:

(27) $$C_{ij}=\frac{\sum_{k=1}^{n}\left(X_{k,i}-\bar{X_{i}}\right)(X_{k,j}-\bar{X_{j}})}{\sqrt{\sum_{k=1}^{n}{X_{k,i}-\bar{X_{i}}}^{2}}\sqrt{\sum_{k=1}^{n}{\left(X_{k,j}-\bar{X_{j}}\right)}^{2}}}$$

where $C_{ij}$ is the correlation coefficient between the timing information of node i and that of node j.

After that, the time series data information of target node i is changed, and the time series data of node i after the change is as follows.

(28) $$X_{i}^{\prime }=X^{max}+X^{min}-X_{i}$$

where $X^{max}\;and\;\;X^{min},$ respectively, are the maximum and minimum values of the timing data information of the target node i in the time window. The information is changed after the target node, and the correlation between neighbor nodes is changed in front of the target node. The correlation between neighbor nodes is now different, based on the data injected with spatial correlation exception information.

- Time correlation between abnormal data.

On the same sensor node, the temporal information of different modalities has a temporal correlation. First, the different modal window temporal correlation information of the same node is calculated in the same period of time. The corrcoef function is used to process the pair of time series data. When the absolute value of the result is closer to 1, the degree of correlation is higher. On the contrary, when the absolute value of the result is closer to 0, the degree of correlation is lower. Then, the timing data information is changed, and the changed timing data are as follows:

(29) $$X_{t,\;\;t+\tau }^{\prime }=X_{t,\;\;t+\tau }-2\times (X_{t,\;\;t+\tau }-X_{t})$$

The correlation coefficient between the modes after the change is calculated and compared with the correlation coefficient before the change. If the correlation coefficient between the modes is not the same, the temporal correlation between the modes of the WSN is changed. That is to say, the data injected with abnormal temporal correlation information are obtained.

#### 3.6.3. Evaluation Index

The model evaluation metrics selected in this section are precision, recall, and the F1 score.

In the classification problem, there are two types of samples in the original data: normal samples and abnormal samples. After the classification model is trained, there will be two results: the model prediction is correct and the model prediction is wrong. The number of different classification results of the model can be recorded by counting. Therefore, four classification model evaluation indicators, including TP, TN, FP, and FN, are set. They represent the true class (TP), which is predicted as normal samples and is actually normal samples; the true negative class (TN), which is predicted as abnormal samples and is actually abnormal samples; the false positive class (FP), which is predicted as normal samples but is actually abnormal samples; and the false negative class (FN), which is predicted as abnormal samples but is actually normal samples. TP + FN represent the actual number of normal samples, TN + FP represent the actual number of abnormal samples, TP + FP represent the model prediction for the number of normal samples, and TN + FN represent the model prediction for the number of abnormal samples.

The relevant probabilities can be calculated for training the model performance evaluation.

Precision is the proportion of normal samples that are correctly predicted to be normal. Precision reflects the accuracy of the model in the case of normal samples, which is more suitable for the scenario of focusing on the classification results of a certain class. Its computation formula is:

(30) $$Precision=\frac{TP}{TP+FP}$$

Recall differs from precision in that recall focuses more on the proportion of the normal sample that is successfully predicted. In classification scenarios where misses have a significant impact on risk, more attention is often paid to recall. It is calculated as follows.

(31) $$Recall=\frac{TP}{TP+FN}$$

When precision and recall are in conflict, we need a more balanced metric to evaluate model performance. The F1 score can be designed by combining precision and recall. The core idea of the F1 score is to maximize precision and recall while keeping the difference between them as small as possible. It can be seen from the following formula that the F1 score is positively correlated with precision and recall.

(32) $$F1=\frac{2Precision\times Recall}{Precision+Recall}$$

#### 3.6.4. Ablation Experiments

**Figure 9:**
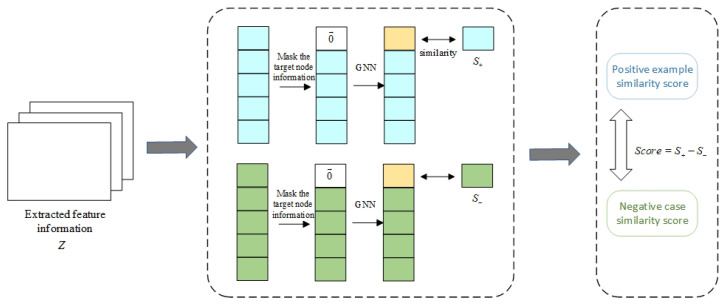
Similarity score calculation module structure diagram.

In order to verify the performance and effect of the metric learning method and feature extraction method designed in this paper, we conducted ablation experiments for these two modules. The specific research contents are as follows:

In order to prove the superiority of the distance-measuring module, a similarity score module was designed to compare the degree of similarity between the normal and abnormal samples. To obtain a similar score, the module learns the target node information of the original target node, striving to obtain the similarity degree of the two scores, and, finally, obtains the positive case of the sample similarity. The negative case of the sample similarity is an unwanted outcome. The module structure is shown in Figure 9.

The feature extraction module for normal and abnormal information begins with the input information module. To prevent the target node’s information from affecting that of other nodes, we first preprocessed the positive and negative examples by masking the target node’s original data. In this case, we used a zero vector to replace the original information of the target node $v_{i}$:

(33) $$\begin{array}{c} X_{i}[i:]=\vec{0}\\ X_{mask}=X_{i} \end{array}$$

Secondly, we used a GCN to learn the information expression of node $v_{i}$:

(34) $$H_{i}=GCN(A,X_{mask},W)$$

where W represents the weight matrix of the GCN iteration layer. Additionally, from the initial data, we extracted the information of the node $v_{i}$ itself:

(35) $$X_{i}=X[i:]$$

Next, we employed a bilinear layer to create a contrast module that learns the similarity score between the node $v_{i}$ and the information of $v_{i}$, which was obtained by aggregating data from the other nodes:

(36) $$S_{i}=Bilinear(X_{i},H_{i})=\sigma (X_{i}\cdot W_{i}\cdot H_{i})$$

where $W_{i}$ is the learnable weight matrix and is the activation function. Finally, the anomaly score was obtained by subtracting the similarity scores of the positive and negative examples. When training with the module of the model, the basic principles narrow the distance when samples belong to the same sample; when samples belong to different categories, the distance between the samples is enlarged, and the similar samples are placed as far away as possible.

This paper presents four proposed schemes. The first scheme utilizes a GCN-based feature extraction method to extract graph data features, followed by a similarity score calculation module to obtain the similarity score and perform anomaly detection. In the second scheme, the similarity score calculation module is replaced with a distance measurement module, enabling anomaly detection through metric learning. The third scheme introduces a feature extraction method combining GCN, GAT, and Transformer modules. It uses the similarity score to fully extract feature information from different categories of samples and detect anomalies. The fourth scheme combines deep learning and metric learning with temporal feature information for anomaly detection.

Table 2 presents a comparison of the model performance between the anomaly detection approach using the similarity score calculation module and the WSN anomaly detection framework (ST-DMAD) with temporal and spatial features, based on deep metric learning, introduced in this study. From the analysis of the experimental results, it can be concluded that the feature extraction method using the GCN outperforms the Transformer-based feature extraction module in capturing more information related to temporal and spatial correlations in the complete graph data. The experimental results show that the F1 score ratio of the three schemes improved by 21%, and the F1 score of the four schemes increased by 4% in the scheme 2 phase. This indicates that the feature extraction module proposed in this paper is more efficient. Based on the similarity information acquisition method of comparison, the F1 score of plan 2 rose by 24%, while the F1 score of plan 4 compared with the F1 score of plan 3 rose by 7%, suggesting that the use of the positive and negative case samples in the measurement learning method can more accurately obtain the similarity between sample information. Compared with the similarity score calculation module, which only considers the similarity difference between positive samples and negative samples, the distance measurement module can more comprehensively consider the similarity relationship between samples from the same class and samples from different classes and make a significant contribution to improving the performance of the model.

#### 3.6.5. Comparative Experiments

This study selected the scheme and put forward the following four algorithms based on the comparative study and reconstruction mechanism of the abnormal WSN node detection method and model. The design scheme is compared in this section.

- CNN-LSTM.

This approach [20] primarily employs the Conv2D convolution module, ReLU activation function, and the MaxPool2D pooling layer with the largest size, along with both short-term and long-term memory components provided by the artificial neural network (LSTM) layer, all within the model framework’s fully connected structure. In the feature extraction phase, the framework of the feature extraction module is built using the following structure: Conv2D-MaxPool2D-ReLU-Conv2D-MaxPool2D. The features extracted are then fed into the LSTM network, where the input parameters, including the hidden layer state and unit state, are initialized. After LSTM, the characteristic information output by LSTM and, finally, a layer of hidden layers and the unit state are obtained. Through full connection via the double-layer classification, the CNN is capable of extracting valuable feature information from local data, particularly excelling at learning hidden features. On the other hand, LSTM offers long-term memory capabilities, effectively addressing the issue of long-term dependencies that traditional RNNs face.

- GCN-LSTM.

This scheme designs an anomaly detection model framework [40] combining GCNs and LSTM networks. In the framework, the GCN can learn the characteristics of the WSN’s topology information. The GCN is used to build a characteristic information extraction module, and then the LSTM is used to handle multiple node GCN extraction modal characteristics of temporal information. This approach eliminates the need to create multiple branches for handling multi-node and multi-modal scenarios, thus avoiding the issue of increased training costs associated with larger model sizes.

- GAT-GRU.

To capture the multi-node and multi-modal time series data feature information of the WSN, the approach in [41] develops modules for extracting sensor node spatial position features, modal correlation features, and time series data features. The detailed model framework is as follows: First, the WSN data are organized according to the nodes, and then, the time series data from each node are input into both the modal correlation feature extraction module and the time series data feature extraction module simultaneously. The modal characteristic information and time series data feature information are combined, and the relevant feature information is studied using a graph attention network (GAT). The key distinction is that the modal correlation feature extraction module calculates the correlation coefficient between different modes of the time series data using a formula, while the time series data feature extraction module employs a matrix of ones as the adjacency matrix. The assumption is that there is a connection relationship between all nodes, and we use map network attention for the attention mechanism between the data information of autonomous learning for different time attention weight coefficients. Next, the data from adjacent time points is aggregated based on the attention weight coefficients. The multi-modal time series data features from multiple nodes are then combined and used as input. The sensor node spatial location feature extraction module employs the matrix to capture the WSN’s topology information and learns the spatial relationships between the nodes.

- GAT-Transformer.

This approach [27] leverages a graph attention network and Transformer to create an anomaly detection model. The process involves several steps: initially, a heterogeneous information network is constructed using real-world datasets, where nodes and the relationships between them are mapped onto the nodes and edges of a graph. Essentially, the heterogeneous information network is treated as graph data. Next, a combination of graph convolutional networks and a non-negative distance matrix is employed to learn the similarities within the graph data. The non-negative distance matrix decomposes the low-dimensional output of the GCN into two status matrices, which helps address the model’s overfitting issue. Once the similarity grouping results are obtained through MLP, the method integrates the GAT and Transformer to extract the relationships between various variables in the graph data. The GAT is designed to capture spatial correlations, while the Transformer is used to embed contextual or temporal correlation information. This approach emphasizes the spatio-temporal correlation in time series data, along with key location-specific details that are often overlooked, effectively integrating similarity information with feature extraction. Unlike traditional clustering methods, the proposed scheme is capable of capturing more comprehensive correlation information.

Table 3 presents the comparative experimental results of the various methods, including the recall rate, precision, and F1 score for the four schemes, with a focus on the WSN anomaly node detection method based on contrastive learning and the reconstruction mechanism. The performance of the proposed space–time feature WSN anomaly detection framework (st-dmad), which utilizes deep metric learning, outperforms that of CNN-LSTM. Specifically, the accuracy, recall rate, and F1 score of the st-dmad model are 20%, 24%, and 22% higher than those of CNN-LSTM, respectively. This improvement is due to CNN-LSTM’s failure to fully leverage the spatial and temporal correlations within the WSN, its inability to extract feature information from graph data comprehensively, and its neglect of the influence of neighboring and related information between target nodes. In contrast, the graph neural network (GNN) is better at extracting complex features from graph data, effectively capturing both temporal correlations in time series data and spatial correlations across multiple nodes, which significantly enhances the model’s feature extraction capabilities. Additionally, GNNs are particularly effective at handling high-dimensional data.

GCN-LSTM can effectively extract the spatial correlation feature information of WSN time series data by using the GCN. However, the GCN uses the complete adjacency matrix in calculations when transferring between layers, which makes the GCN need more cost to update the full graph information when processing large-scale data, and it is prone to the problem of model overfitting. GAT-GRU, GAT–Transformer, and ST-DMLAD can better extract the correlation information between the target node and its neighbor nodes and complete the parallel calculation without the need for a fixed sampling window size, which improves the efficiency of the model. Compared with GCN-LSTM, the F1 scores of GRU, GAT-Transformer, and ST-DMLAD are improved by 9%, 5%, and 13%, respectively.

GAT-GRU divides the time series data of the WSN into branches from the perspective of nodes, which means the model needs to add a new branch to learn the relevant information expression of the node after adding a new node. When there are a large number of nodes in the WSN, the size of the model will increase, which greatly affects the cost of training the model. Therefore, using the spatial feature extraction module to extract the corresponding spatial features can avoid the problem of too many branches.

GAT–Transformer combines a GCN with a non-negative distance matrix to learn the similarity of graph data, whereas the metric learning method more accurately captures the similarity relationships and autonomously learns the approach to determine similarity. Additionally, GAT–Transformer only focuses on the similarity between normal and abnormal samples, whereas ST-DMLAD takes into account not only the similarity between samples of different classes but also the influence of similarity within positive samples and between negative samples. Compared with the experimental results of GAT–Transformer, the recall rate of ST-DMLAD increased by 13%, and the F1 score increased by 8%.

### 3.7. Summary of This Section

In this section, we introduce an anomaly detection framework for WSNs that integrates metric learning with deep learning techniques. To comprehensively capture the multi-node and multi-modal time series features in WSNs, we design a fusion spatio-temporal feature extraction module. This module leverages graph attention networks (GATs) and Transformers to efficiently extract both spatial and temporal correlation information, aiding in the effective feature extraction of multi-node and multi-modal time series data. To address the challenge of a limited number of samples, which prevents the establishment of a parameterized mapping from features to categories, this paper integrates the strengths of metric learning and deep learning. Metric learning enables the model to determine whether samples belong to the same class based on feature distances, while deep learning effectively extracts feature representations in WSNs. This combination enhances the model’s ability to differentiate between normal and anomalous samples.

Additionally, a joint training approach incorporating both metric learning and classification is employed to maximize the utilization of label information within the dataset. To thoroughly account for intra-class and inter-class similarity, the proposed distance measurement module adopts the triplet loss function. By introducing positive and negative sample pairs, the model ensures that positive samples are positioned closer to the target sample, while negative samples are pushed farther away. This design facilitates more effective learning of similarity relationships. Furthermore, the training cost and computational complexity of the model can be further optimized.

## 4. Conclusions

### 4.1. Summary of the Main Research Work

WSNs, with unlimited communication and multi-hop routing, can greatly reduce the security and reliability of a network. These sensors may fail at any given point in time, or intruders may attack the nodes, thus deteriorating the network and causing problems in collecting data from sensors. Especially in the process of information transmission, they are vulnerable to external interference and intrusion, resulting in information leakage and network structure damage, and malicious nodes may even be inserted to make the network unable to obtain the corresponding information. The characteristics of wireless communication of WSNs determine that the privacy of the wired network cannot be obtained. With the widespread use of WSNs in more practical fields, WSN-related network data security issues must be paid more and more attention.

The main research work of this paper is summarized as follows:

To address the limitations of the existing methods in learning accurate mappings from features to categories and capturing similarity between normal and abnormal samples in small-sample scenarios, this paper proposes a novel approach. We employ a GAT and an optimized Transformer to extract spatio-temporal correlations in multi-node, multi-modal WSN time series data, enhancing feature representation. Then, metric learning is applied to model sample similarity using both positive and negative sample pairs to capture intra-class and inter-class relationships effectively.

### 4.2. Prospects for Future Research

This paper provides some insight into the field of wireless sensor anomaly detection, and the directions that can be explored in future research are as follows:

(I) There are a lot of application scenarios of dynamic graphs in reality. The data volume of dynamic graphs is much larger than that of static graphs, and the data complexity is higher. Moreover, dynamic graph data are difficult to obtain and are noisy, redundant, and high-sparsity. At the same time, they have high research value.

(II) In this paper, the imbalance of data samples, the diversity of sample information feature extraction, and spatio-temporal correlation features are discussed, but the work basically belongs to the discussion of time domain characteristics of data. For time series data collected by sensors, it is also effective to analyze local information and global information from the perspective of the frequency domain. Identifying how to perform data augmentation and extract various correlation features in frequency domain analysis remains an important challenge. In addition, designing updated generative model methods, especially by combining them with emerging large-model generative AI techniques, is crucial. These directions hold significant research value for advancing information extraction strategies in the field of WSN anomaly detection.

(III) In practical application scenarios, each data sample of each variable in time series data can be regarded as a dimension. With the increase in the dimension of time series data, the size of the data space will grow exponentially, which makes the data information become sparse. There are a large number of missing values in sparse data, which makes the data information extremely incomplete. These data are widely used in applications such as e-commerce, medical imaging, questionnaires, and telephone surveys. Clustering and dimensionality reduction for sparse data is a promising research direction.

## Figures and Tables

**Table 1:** Module package versions.

Module Package	Version Number
datashape	0.5.4
matplotlib	3.5.2
matplotlib-inline	0.1.6
numpy	1.21.5
pandas	1.4.4
pip	22.2.2
scipy	1.9.1
torch	1.13.0
torchvision	0.14.0
tqdm	4.64.1
wheel	0.37.1
zipp	3.8.0

**Table 2:** Ablation experiment results.

Serial Number	Feature Extraction Method		Similarity Acquisition Method		Prec	Rec	F1
	GCN	This Feature Extraction Module	Similarity Score Calculation Module	This Design Distance Measurement Module			
1	√		√		0.72	0.53	0.61
2	√			√	0.97	0.76	0.85
3		√	√		0.8	0.83	0.82
4		√		√	0.95	0.84	0.89

**Table 3:** Comparison of experimental results.

Option	Prec	Rec	F1
CNN-LSTM	0.75	0.6	0.67
GCN-LSTM	0.79	0.73	0.76
GAT-GRU	0.89	0.82	0.85
GAT–Transformer	0.95	0.71	0.81
**ST-DMLAD**	**0.95**	**0.84**	**0.89**

## Data Availability

The data used in this study are from the publicly available IBRL dataset, which can be accessed at http://db.csail.mit.edu/labdata/labdata.html, accessed on 1 March 2025.
